# Fatty acid composition of blood plasma in multiple organ dysfunction syndrome

**DOI:** 10.1186/cc13292

**Published:** 2014-03-17

**Authors:** AN Osipenko, Av Marochkov

**Affiliations:** 1 A. Kuleshov Mogilev State University, Mogilev, Belarus; 2 Mogilev Regional Hospital, Mogilev, Belarus

## Introduction

The aim of study was to assess the fatty acid (FA) composition of blood plasma in multiple organ dysfunction syndrome (MODS).

## Methods

The objects of the study were 18 people with multiple organ failure (35.6 ± 8.7 years) of various etiologies. The blood of 16 healthy volunteers aged 37.7 ± 3.2 years served as control. There were also analyzed blood plasma and fragments of artery luminal part from patients who died from different causes. Analysis of FA was conducted using capillary gas-liquid chromatography. Quantitative evaluation of individual FA content was made as a mass percentage of their total. Statistical analysis was performed using the Mann-Whitney U test (*P *< 0.05).

## Results

Normalized contents of oleic and palmitoleic monounsaturated FA increase in patients with MODS, while the levels of stearic saturated FA and polyunsaturated FA decrease, as compared with the control. Considering these results, we conclude that blood plasma FA composition in MODS biased towards composition of FA characteristic of adipose and muscle tissue triglycerides, which quantitatively predominant monounsaturated FA, and stearic and polyunsaturated FA have a significantly lower level [1],[2]. Thus, the composition of the FA in MODS reflects the degree of lipolysis activation in fat depots. As a result of these processes there should occur an imbalance in vascular endothelial cells between monounsaturated and polyunsaturated FA and, as a consequence, changes in metabolism of eicosanoids and other lipid vasoactive mediators should develop. One should note that in plasma of people who died in a hospital environment due to various reasons, similar changes in FA blood composition are observed. The degree of change of FA composition in postmortem blood samples presumably primarily depends on severity and duration of a previous critical and serious condition. Postmortem changes presumably exert some influence on blood plasma FA composition. Moreover, the composition of FA in blood plasma in the case of MODS is similar to that of FA in the luminal part of the artery wall. This suggests a decrease in the influence of intertissue differences in lipid composition on metabolic processes.

## Conclusion

Given that the monounsaturated FAs in the case of MODS enter the bloodstream primarily from adipose and muscle tissues, one can assume that their blood plasma level reflects the degree of hypermetabolism processes in the organism.
